# Genetic association between circulating selenium level and the risk of schizophrenia in the European population: A two-sample Mendelian randomization study

**DOI:** 10.3389/fnut.2022.969887

**Published:** 2022-08-23

**Authors:** Ming-Gang Deng, Han-Tao Cui, Jia-Qi Nie, Yuehui Liang, Chen Chai

**Affiliations:** ^1^Department of Epidemiology, School of Public Health, Wuhan University, Wuhan, China; ^2^Department of Nutrition and Food Hygiene, School of Public Health, Wuhan University, Wuhan, China; ^3^Emergency Center, Hubei Clinical Research Center for Emergency and Resuscitation, Zhongnan Hospital of Wuhan University, Wuhan, China

**Keywords:** nutrients, selenium, schizophrenia, Mendelian randomization, European population

## Abstract

**Background:**

The association between circulating the selenium level and the risk of schizophrenia remains unclear.

**Objective:**

To determine the relationship between the circulating selenium level and the risk of schizophrenia, using the Mendelian Randomization method in the European population.

**Methods:**

Single nucleotide polymorphisms (SNPs) associated with the circulating selenium level were identified at *p* < 5 × 10^−8^. The inverse variance weighted (IVW) method was used as the principal MR analysis, and MR Egger, weighted median, and MR PRESSO were used to determine the accuracy of IVW results. The Cochran's Q-test and Leave-One-Out sensitivity analysis were performed to evaluate the heterogeneity and stability of genetic variants on schizophrenia.

**Results:**

The circulating selenium level was associated with decreased risk of schizophrenia by the IVW method (OR: 0.906, 95% CI:0.867–0.947). MR Egger, weighted median, and MR PRESSO methods got similar results. No heterogeneity was detected by the Cochran's Q-test, and no single SNP was driving the overall effect by leave-one-out analysis.

**Conclusion:**

Our study provides support for the genetic relationship between the circulating selenium level and schizophrenia; the decreased circulating selenium level was associated with an elevated risk of schizophrenia.

## Introduction

Schizophrenia is a complex neurodevelopmental disorder with a typical onset during late adolescence or early adulthood (1). Symptoms can be classified into three categories, such as positive (e.g., hallucinations, delusions), negative (e.g., social withdrawal, alogia, and flat affect), and cognitive deficiencies (2). The prevalence of schizophrenia in the European region was approximately 328 prevalent cases per 100,000 in 2019 (3). Although the prevalence of schizophrenia is relatively low, individuals living with schizophrenia had a significantly reduced life expectancy (4). High excess mortality was found across all age groups between those with and without schizophrenia (5), and this differential mortality gap may have increased in recent decades (6).

The risk factors of schizophrenia are multiple, including genetic susceptibility and environmental factors (7). Numerous essential mental elements are proved to be associated with the pathophysiology of schizophrenia (8). Selenium is an essential trace element that is incorporated into 25 selenoproteins (9), and is found to be associated with the pathogenesis of schizophrenia by oxidative stress (10, 11).

Nevertheless, the results of observational studies are controversial. For example, the concentration of selenium was significantly reduced in patients with schizophrenia (10, 12). An intervention study claimed that the serum selenium level was lower in patients with schizophrenia than in healthy individuals, and, after selenium supplementation, the patients with schizophrenia improved appetite and memory (13). However, no difference was also obtained between patients with schizophrenia and healthy controls (14–16). Selenium as selenocysteine becomes incorporated into selenoproteins. Selenium binding protein 1 (SELENBP1) was an important selenoprotein and was suggested that genetic variation in SELENBP1 may influence the risk for schizophrenia (17). A study of patients during their first hospitalization with schizophrenia did not report different levels of SELENBP1 mRNA in blood compared with controls (18). However, both downregulation and upregulation of plasma SELENBP1 in schizophrenia patients have also been reported in the other two studies (19, 20). Hence, the exact link between the selenium level and schizophrenia remains unclear.

As a method to use genetic variation to determine whether the observed association between the risk factor and the outcome is consistent with causal effect, Mendelian randomization (MR) can overcome the limitations of observational studies, such as confounding bias and reverse causation (21) and provide more favorable evidence to explore the association between exposure and an outcome (22).

Therefore, we attempt to use the Mendelian Randomization method to determine the relationship between circulating the selenium level and schizophrenia, using the genome-wide association study (GWAS) data from European ancestry.

## Methods

### Selection of instrumental SNPs

We selected instrumental SNPs associated with circulating the selenium level from a GWAS meta-analysis study in 4,162 American adults (23) at the significance level of *p* < 5 × 10^−8^. The association test was adjusted for age, sex, and smoking status (23). Supplementary SNPs, which were strongly associated with the blood selenium level (*p* < 5 × 10^−8^), were selected from a GWAS meta-analysis of 2,603 Australian twins and their families (with adjustment for age, sex, and relatedness) and 2,874 British pregnant women (24).

SNPs were pruned for linkage disequilibrium at *r*^2^ <0.3 (region size = 1,000 kb), and the SNP with the lowest *P-value* for the genome-wide association was retained. SNPs that were unavailable in the outcome datasets were replaced by suitable proxy SNPs that minimum linkage disequilibrium *R*^2^ = 0.8 and a minor allele frequency threshold =0.3, where available. Moreover, we used the PhenoScanner approach (25) to assess whether the IVs were associated with confounders or risk factors in disease (see Supplementary Table S1). Finally, nine SNPs are included in our analysis, which can explain about 12.1% variance of circulating selenium, and detailed information is presented in Table 1. All SNPs were based on HG19/GRCh37, eight SNPs located in gene *DMGDH*, and one SNP located in gene *ARSB*. These SNPs were also mainly associated with other traits, such as height or sitting height, and comparative height size at age 10 (see Supplementary Table S1), which were deemed not to confound the genetic association between the blood selenium level and the risk of schizophrenia.

**Table 1:** Characteristics of instrumental SNPs for circulating the selenium level and schizophrenia.

**SNP**	**Chr**	**Pos**	**MAF**	**Gene**	**EA**	**OA**	***R^2^***	***F***	**Exposure**				**Outcome**		
									**Beta**	**SE**	***P***		**Beta**	**SE**	***P***
rs10514151	5	78303487	0.06	DMGDH	T	C	0.005	20.599	0.209	0.059	1.42E−08		−0.023	0.0222	2.97E−01
rs163124	5	78283003	0.28	ARSB	G	T	0.009	37.067	0.148	0.034	5.16E−12		−0.012	0.0122	3.15E−01
rs16876498	5	78402594	0.10	DMGDH	C	T	0.017	72.268	0.308	0.048	1.23E−22		−0.052	0.018	3.52E−03
rs3797535	5	78300397	0.10	DMGDH	T	C	0.008	34.252	0.213	0.057	2.42E−09		−0.022	0.0205	2.89E−01
rs478651	5	78290682	0.48	DMGDH	T	C	0.013	55.224	0.162	0.035	3.53E−13		−0.002	0.0111	8.69E−01
rs586199	5	78405090	0.50	DMGDH	G	A	0.02	85.767	0.201	0.029	2.37E−26		−0.023	0.011	3.25E−02
rs705415	5	78291960	0.88	DMGDH	C	T	0.011	47.833	0.232	0.059	4.56E−10		0.007	0.0186	6.99E−01
rs921943	5	78316476	0.30	DMGDH	T	C	0.025	142.786	0.246	0.034	9.4E−28		−0.030	0.0119	1.25E−02
rs949644	5	78442351	0.67	DMGDH	A	G	0.012	51.944	0.167	0.031	2.03E−16		−0.019	0.0113	9.10E−02

*SNP, single-nucleotide polymorphism; Chr, chromosome; Pos, position based on GRCh37/hg19; MAF, minor allele frequency; EA, effect allele; OA, other allele*.

### Genetic associations with the outcomes

Summary statistics of schizophrenia were obtained from a previous GWAS study (26), conducted by the *Schizophrenia Working Group of the Psychiatric Genomics Consortium* in 2014. The study included 33,640 cases and 43,456 controls, where genome-wide genotype data were obtained from 49 European ancestries matched, non-overlapping case-control samples, and three family-based samples of European ancestry (1,235 parent-affected offspring trios). These participants mainly came from England, Sweden, Germany, Finland, and other European countries, and some came from non-European countries, such as America, Canada, etc. More detailed information could be found at https://www.nature.com/articles/nature13595#Sec9.

### Statistical analysis

The genetic IV-risk factor association and genetic IV-outcome association in our study are generated from different (largely non-overlapping) countries of European descent. According to the MR principles (27), we used the two-sample MR analysis to evaluate the relationship between circulating the selenium level and the risk of schizophrenia.

The inverse variance weighted (IVW) approach was adopted as the primary MR analysis to assess the potential effect of selenium on the risk of schizophrenia. Moreover, MR Egger and weighted median methods were supplemented to improve the robustness of estimates.

The IVW method assumes the absence of average pleiotropic effect, and, in this case, it is the most efficient method (28). The MR Egger method is robust to pleiotropy and sensitive to outliers, and it gives an unbiased estimation if genetic pleiotropy is present (29). The weighted median method assumes that at least 50% of IVs are effective, which orders the MR estimates of each IV weighted for the inverse of their variance (30). Thus, this method is robust to outliers and sensitive to the addition or removal of genetic variants.

Several sensitivity analyses were conducted to evaluate the robustness of our main analyses. The MR pleiotropy residual sum and outlier (MR-PRESSO) global test method was conducted to detect horizontal pleiotropy, and the MR-PRESSO outlier test was conducted to correct the horizontal pleiotropy *via* outlier removal. The Cochran's Q-test and “leave-one-out” sensitivity analysis were performed to evaluate the heterogeneity and stability of these genetic variants on schizophrenia.

The TwoSampleMR (version 0.5.6) and MRPRESSO (version 1.0) packages were used for statistical analyses in R software (version 4.1.0, R Foundation for Statistical Computing). All statistical tests were two-tailed, and significance was considered at a Bonferroni-corrected *p*-value <0.025 (correcting for two exposures and one outcome).

## Results

### Blood selenium level and schizophrenia

**Figure 1:**
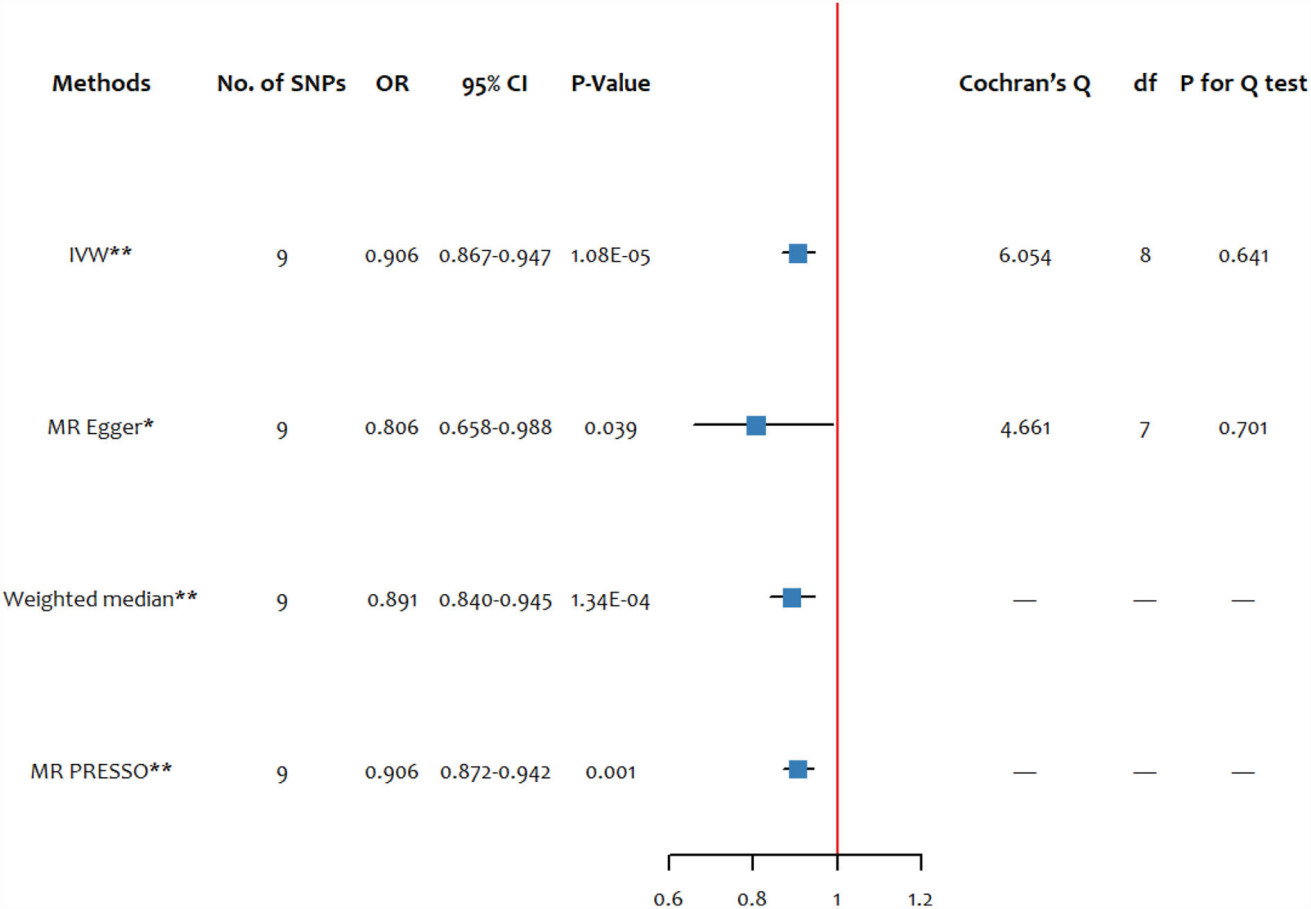
The forest plots of MR estimates and the Cochran's Q-test for the blood selenium level on schizophrenia. SNP, single-nucleotide polymorphism; df, degree of freedom; IVW, inverse variance weighted; MR-PRESSO, MR pleiotropy residual sum and outlier. ^*^ significant at *p* < 0.05, ^**^ significant at *p* < 0.025.

The MR estimates of selenium on schizophrenia and the Cochran's Q-test are presented in Figure 1. Our analysis supported a potential relationship between the blood selenium level and schizophrenia by the IVW method (OR: 0.906, 95% CI: 0.867–0.947). Sensitive analysis conducted by MR Egger and weighted median methods got more significantly protective results.

The MR PRESSO global test did not find any outliers in the SNPs of schizophrenia, and the statistic and *P-*value were 7.813 and 0.694, respectively. The MR PRESSO estimate of the effect of the blood selenium level on schizophrenia was similar to the main analysis, showing a protective effect (OR: 0.906, 95% CI: 0.872–0.942).

There was no evidence for heterogeneity measured by the Cochran's Q test (*Q*_IVW_ = 6.054, *P*_IVW_ =0.641; *Q*_Egger_ = 4.661, *P*_Egger_ =0.701). In our study, no pleiotropic effects were found by the MR Egger regression intercept (intercept =0.021, *p* = 0.288).

**Figure 2:**
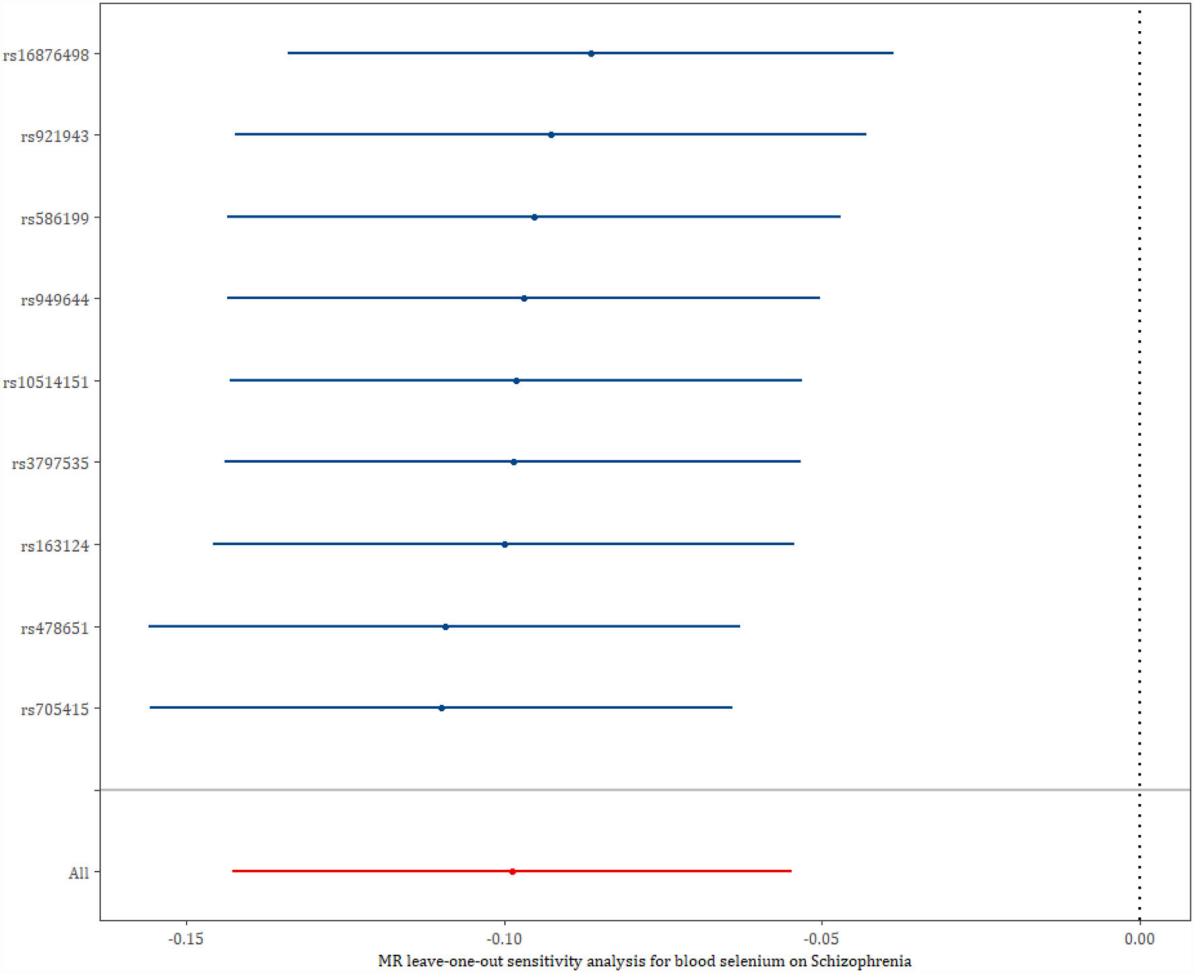
The leave-one-out analysis of genetic variants on schizophrenia.

The result of the leave-one-out analysis of genetic variants of the blood selenium level on schizophrenia is presented in Figure 2. The leave-one-out analysis indicated that there was no single SNP driving the overall effect, and it suggested the stability of our results.

The scatter plot of the effects of SNPs on the blood selenium level and schizophrenia was illustrated in Supplementary Figure S1. The forest plot combing the single SNP effect estimates of the blood selenium level on the risk for schizophrenia is displayed in Supplementary Figure S2. Among the nine SNPs, three SNPs' single-effect estimates (rs586199, rs921943, and rs16876498) were significant for the risk of schizophrenia and correlate with changed blood selenium levels. The other SNPs, e.g., rs705415, rs478651, rs163124, etc., were not significant for the risk of schizophrenia and correlate with changed blood selenium levels.

### Sensitivity analysis

Furthermore, we also used an additional IV set associated with blood and the toenail selenium level to assess the robustness of our findings in the sensitivity analysis. Two SNPs (rs234709, rs6586282), associated with homocysteine, which has been confirmed to increase the risk of schizophrenia (31), were excluded from our study (see Supplementary Table S2). The characteristics of blood and the toenail selenium level-associated SNPs on schizophrenia are presented in Supplementary Table S3.

**Figure 3:**
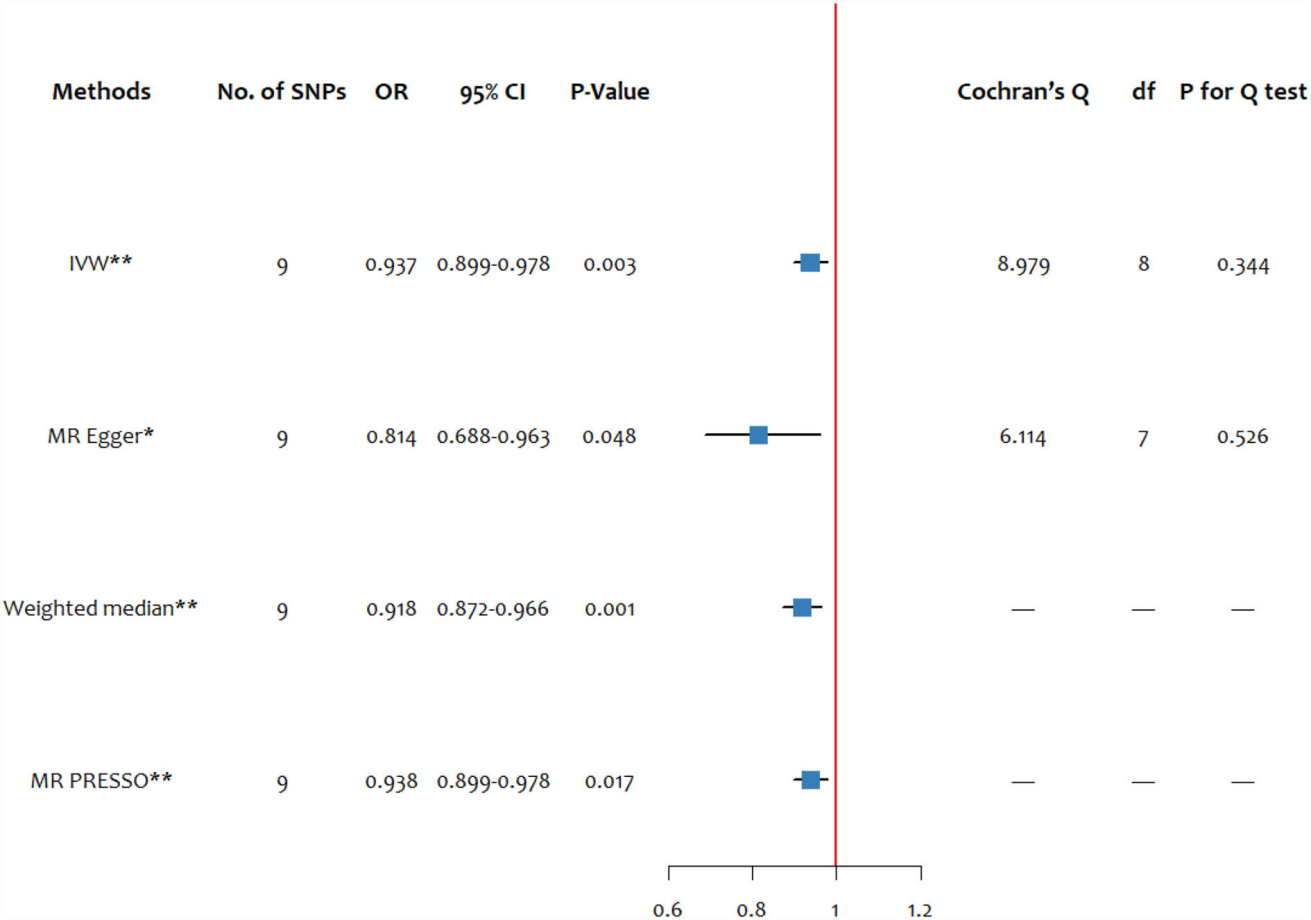
The forest plot of MR estimates and the Cochran's Q-test for blood and the toenail selenium level on schizophrenia. SNP, single-nucleotide polymorphism; df, degree of freedom; IVW, inverse variance weighted; MR-PRESSO, MR pleiotropy residual sum and outlier. ^*^ significant at *p* < 0.05, ^**^ significant at *p* < 0.025.

In the sensitivity analysis of blood and the toenail selenium level-associated SNPs on schizophrenia, the effect remained consistent with those of the blood selenium level (OR: 0.937, 95% CI: 0.899–0.978) (see Figure 3). There was no evidence for heterogeneity measured by Cochran's Q-test (*Q*_IVW_ = 8.979, *P*_IVW_ = 0.344; *Q*_Egger_ = 6.114, *P*_Egger_ = 0.526). No pleiotropic effects were found by the MR Egger regression intercept (intercept =0.034, *p* = 0.134).

The scatter plot of the effect estimates of IVs on blood and toenail selenium and the risk of schizophrenia are presented in Supplementary Figure S3. The forest plot combing the single-SNP effect estimates of blood and the toenail selenium level on the risk of schizophrenia is presented in Supplementary Figure S4. The leave-one-out analysis of genetic variants of blood and toenail selenium level on schizophrenia is presented in Supplementary Figure S5.

### Evaluation of the assumptions of MR

Three critical assumptions must be met for causal estimations derived from MR analysis to be valid: (1) they associate with the risk factor of interest (the relevance assumption); (2) they are not associated with confounders of the risk factor-outcome association (the independence assumption); (3) they do not affect the outcome except through the risk factor (the exclusion restriction assumption) (32).

Firstly, for the relevance assumption, we selected the SNPs that are associated with circulating the selenium level at genome-wide significance (*p* < 5^*^10^−8^), and the *F*-statistics for each SNP were all > 10, which make the selected SNPs robustly associated with exposures, and unlikely result in weak instrument bias. Additionally, for the independence assumption, as mentioned in the methods section, we used the PhenoScanner approach to assess whether the SNPs were associated with confounders or risk factors in disease. The selected SNPs were associated with height or sitting height, and comparative height size at age 10, which were deemed impossible to confound the genetic association between the blood selenium level and the risk of schizophrenia. Lastly, the exclusion restriction assumption may be violated by horizontal pleiotropy. To make our MR analysis meet this assumption, we adopted the Clumping technique to prune for linkage disequilibrium to make the SNPs independent from each other. Moreover, no pleiotropic effects were detected by the MR Egger regression intercept (Intercept_blood selenium_ =0.021, *P*_blood selenium_ =0.288, Intercept_blood and toenail selenium_ =0.034, *P*_blood and toenail selenium_ =0.134).

## Discussion

Using two-sample Mendelian randomization, we found that the circulating selenium level was associated with the risk of schizophrenia in the European population.

In our study, the blood selenium level was associated with a decreased risk of schizophrenia, which was consistent with the previous observational studies (8, 10, 13). These studies concluded that a lower concentration of selenium was significantly associated with an elevated risk of schizophrenia. Furthermore, another two clinical trials provided more robust evidence that selenium supplementation had beneficial, statistically significant effects on schizophrenia, such as the general Positive and Negative Syndrome Scale (PANSS) score, metabolic profiles (33), appetite, and memory (13).

Mechanistically, there were several potential explanations. Oxidative stress was involved in schizophrenia pathogenesis (11, 34), and at least half of selenoproteins participate in suppressing oxidative stress (9). Dopamine has been proposed to contribute significantly to the pathophysiology of schizophrenia (35), and selenium-dependent glutathione peroxidase plays a protective role against dopaminergic toxicity induced by methamphetamine (36). Additionally, fatty acids reported to have a significantly negative association with selenium (10) were identified as a potential risk for schizophrenia (37). Moreover, another study suggested that selenium intake in patients with schizophrenia can increase the level of copper and zinc in serum, which can elevate the capability of appetite and memory (13), and depletion of maternal copper can affect fetal development and may leave the offspring prone to schizophrenia (38).

This study provided the genetic evidence that the elevated circulating selenium level was related to decreased risk of schizophrenia by using the MR design, which can minimize biases from residual confounding and reverse causation. Our study was performed under the validation of the three critical assumptions of the MR study. The results were robust enough, which were confirmed by different MR approaches, such as IVW, MR-Egger, weighted median, and MRPRESSO. No heterogeneity was found within the SNPs, and leave-one-out analysis demonstrated that the overall effect was not driven by a single SNP, indicating the stability of our results. The sensitivity analysis by using the blood and toenail selenium-associated SNPs also backed up the beneficial effect of selenium on schizophrenia. Meanwhile, a number of mechanisms, as described above, support the biological plausibility for our research.

Limitations need to be considered when interpreting our findings. We studied the relationship between the selenium level and the risk of schizophrenia, not the selenium level in cerebrospinal fluid or prefrontal cortex, which might lead to different results. Additionally, the non-linear relationship of the selenium level with risk for schizophrenia was not evaluated. Besides, genome-wide association studies about selenium exposure only adjusted for sex, age, and smoking, other potential factors may affect the real association of the selected SNPs with selenium exposure, and thus induce bias. Moreover, the cases were individuals with schizophrenia or schizoaffective disorder. The schizophrenia cases were not classified according to symptoms, such as positive, negative, and cognitive deficiencies. Therefore, the relationship between blood selenium levels and specific symptoms of schizophrenia cannot be derived, as limited by the current GWAS research. Furthermore, we restricted the sample to European ancestry to reduce bias, which might make our results not applicable to other populations.

## Conclusion

Our study provides genetic support for a relationship between the circulating selenium level and schizophrenia; the decreased circulating selenium level was associated with an elevated risk of schizophrenia.

## Data availability statement

The original contributions presented in the study are included in the article/Supplementary material, further inquiries can be directed to the corresponding author/s.

## Author contributions

All authors listed have made a substantial, direct, and intellectual contribution to the work and approved it for publication.

## Conflict of interest

The authors declare that the research was conducted in the absence of any commercial or financial relationships that could be construed as a potential conflict of interest.

## Publisher's note

All claims expressed in this article are solely those of the authors and do not necessarily represent those of their affiliated organizations, or those of the publisher, the editors and the reviewers. Any product that may be evaluated in this article, or claim that may be made by its manufacturer, is not guaranteed or endorsed by the publisher.
