# Supplementary material for: Genetic association between circulating selenium level and the risk of schizophrenia in the European population: A two-sample Mendelian randomization study
Source: Front Nutr. 2022 Aug 23;9:969887. doi: 10.3389/fnut.2022.969887 (PMC9445556; doi:10.3389/fnut.2022.969887)
Supplement: Supplementary file 1 [file Data_Sheet_1.PDF]

Supplementary Material

Supplementary Table S1 The results of PhenoScanner of blood selenium level associated SNPs

Supplementary Table S2 The results of PhenoScanner of blood and toenail selenium level associated SNPs

Supplementary Table S3 The characteristics of blood and toenail selenium level associated SNPs.

Supplementary Figure S1 Scatter plot of the effect estimates of IVs on blood selenium and risk of schizophrenia.

Supplementary Figure S2 Forest plot combing the single SNP effect estimates of blood selenium on the risk of schizophrenia.

Supplementary Figure S3 Scatter plot of the effect estimates of IVs on blood and toenail selenium and risk of schizophrenia.

Supplementary Figure S4 Forest plot combing the single SNP effect estimates of blood and toenail selenium level on the risk of schizophrenia

Supplementary Figure S5 The leave-one-out analysis of genetic variants of blood and toenail selenium level on schizophrenia.

Supplementary Table S1 The results of PhenoScanner of blood selenium level associated SNPs

SNP	EA	OA	trait	study	PubMed ID	ancestry	year	dataset
rs672413	G	A	Blood and toenail selenium levels	Cornelis MC	25343990	European	2014	NHGRI-EBI_GWAS_Catalog
rs672413	G	A	Blood protein levels	Suhre K	28240269	European	2017	NHGRI-EBI_GWAS_Catalog
rs163124	T	G	Comparative height size at age 10	Neale B	UKBB	European	2017	Neale-B_UKBB_EUR_2017
rs163124	T	G	Sitting height	Neale B	UKBB	European	2017	Neale-B_UKBB_EUR_2017
rs163132	T	C	Comparative height size at age 10	Neale B	UKBB	European	2017	Neale-B_UKBB_EUR_2017
rs163132	T	C	Height	Neale B	UKBB	European	2017	Neale-B_UKBB_EUR_2017
rs163132	T	C	Sitting height	Neale B	UKBB	European	2017	Neale-B_UKBB_EUR_2017
rs7700970	C	T	Selenium levels	Evans DM	23720494	European	2013	GRASP
rs7700970	C	T	Toenail selenium levels	Cornelis MC	25343990	European	2014	NHGRI-EBI_GWAS_Catalog
rs16876394	C	T	Blood metabolite levels	Shin SY	24816252	European	2014	NHGRI-EBI_GWAS_Catalog
rs16876394	C	T	Sitting height	Neale B	UKBB	European	2017	Neale-B_UKBB_EUR_2017
rs16876498	C	T	Sitting height	Neale B	UKBB	European	2017	Neale-B_UKBB_EUR_2017
rs17823744	A	G	Toenail selenium levels	Cornelis MC	25343990	European	2014	NHGRI-EBI_GWAS_Catalog
rs17823744	A	G	Height	Neale B	UKBB	European	2017	Neale-B_UKBB_EUR_2017
rs17823744	A	G	Sitting height	Neale B	UKBB	European	2017	Neale-B_UKBB_EUR_2017
rs248380	T	C	Sitting height	Neale B	UKBB	European	2017	Neale-B_UKBB_EUR_2017
rs3797535	C	T	Blood and toenail selenium levels	Cornelis MC	25343990	European	2014	NHGRI-EBI_GWAS_Catalog
rs586199	A	G	Sitting height	Neale B	UKBB	European	2017	Neale-B_UKBB_EUR_2017
rs705415	T	C	Blood and toenail selenium levels	Cornelis MC	25343990	European	2014	NHGRI-EBI_GWAS_Catalog
rs7710824	A	C	Height	Neale B	UKBB	European	2017	Neale-B_UKBB_EUR_2017
rs921943	T	C	Selenium levels	Evans DM	23720494	European	2013	GRASP
rs921943	T	C	Blood and toenail selenium levels	Cornelis MC	25343990	European	2014	NHGRI-EBI_GWAS_Catalog
rs921943	T	C	Blood trace element Se levels	Evans DM	23720494	European	2013	NHGRI-EBI_GWAS_Catalog
rs921943	T	C	Comparative height size at age 10	Neale B	UKBB	European	2017	Neale-B_UKBB_EUR_2017
rs921943	T	C	Height	Neale B	UKBB	European	2017	Neale-B_UKBB_EUR_2017
rs921943	T	C	Sitting height	Neale B	UKBB	European	2017	Neale-B_UKBB_EUR_2017
rs9293761	A	G	Sitting height	Neale B	UKBB	European	2017	Neale-B_UKBB_EUR_2017

SNP, single-nucleotide polymorphism; EA, effect allele; OA, other allele.

Supplementary Table S2 The results of PhenoScanner of blood and toenail selenium level associated SNPs

SNP	EA	OA	trait	study	PubMed ID	ancestry	year	dataset
rs10944	T	G	Blood and toenail selenium levels	Cornelis MC	25343990	European	2014	NHGRI-EBI_GWAS_Catalog
rs10944	T	G	Sitting height	Neale B	UKBB	European	2017	Neale-B_UKBB_EUR_2017
rs11951068	A	G	Blood and toenail selenium levels	Cornelis MC	25343990	European	2014	NHGRI-EBI_GWAS_Catalog
rs1789953	C	T	Blood and toenail selenium levels	Cornelis MC	25343990	European	2014	NHGRI-EBI_GWAS_Catalog
rs234709	C	T	Blood and toenail selenium levels	Cornelis MC	25343990	European	2014	NHGRI-EBI_GWAS_Catalog
rs234709	C	T	**Homocysteine levels**	van Meurs JB	23824729	European	2013	NHGRI-EBI_GWAS_Catalog
rs3797535	C	T	Blood and toenail selenium levels	Cornelis MC	25343990	European	2014	NHGRI-EBI_GWAS_Catalog
rs567754	C	T	Blood and toenail selenium levels	Cornelis MC	25343990	European	2014	NHGRI-EBI_GWAS_Catalog
rs567754	C	T	Toenail selenium levels	Cornelis MC	25343990	European	2014	NHGRI-EBI_GWAS_Catalog
rs6586282	C	T	**Homocysteine levels**	Lange LA	20154341	Filipino	2010	GRASP
rs6586282	C	T	Plasma homocysteine	Pare G	20031578	European	2009	GRASP
rs6586282	C	T	Selenium levels	Evans DM	23720494	European	2013	GRASP
rs6586282	C	T	Blood and toenail selenium levels	Cornelis MC	25343990	European	2014	NHGRI-EBI_GWAS_Catalog
rs6586282	C	T	Blood trace element Se levels	Evans DM	23720494	European	2013	NHGRI-EBI_GWAS_Catalog
rs6586282	C	T	**Homocysteine levels**	Pare G	20031578	European	2009	NHGRI-EBI_GWAS_Catalog
rs6586282	C	T	**Homocysteine**	Pare G	20031578	European	2009	dbGaP
rs672413	G	A	Blood and toenail selenium levels	Cornelis MC	25343990	European	2014	NHGRI-EBI_GWAS_Catalog
rs672413	G	A	Blood protein levels	Suhre K	28240269	European	2017	NHGRI-EBI_GWAS_Catalog
rs6859667	C	T	Blood and toenail selenium levels	Cornelis MC	25343990	European	2014	NHGRI-EBI_GWAS_Catalog
rs6859667	C	T	Toenail selenium levels	Cornelis MC	25343990	European	2014	NHGRI-EBI_GWAS_Catalog
rs6859667	C	T	Rash and other nonspecific skin eruption	Neale B	UKBB	European	2017	Neale-B_UKBB_EUR_2017
rs705415	T	C	Blood and toenail selenium levels	Cornelis MC	25343990	European	2014	NHGRI-EBI_GWAS_Catalog
rs921943	T	C	Selenium levels	Evans DM	23720494	European	2013	GRASP
rs921943	T	C	Blood and toenail selenium levels	Cornelis MC	25343990	European	2014	NHGRI-EBI_GWAS_Catalog
rs921943	T	C	Blood trace element Se levels	Evans DM	23720494	European	2013	NHGRI-EBI_GWAS_Catalog
rs921943	T	C	Comparative height size at age 10	Neale B	UKBB	European	2017	Neale-B_UKBB_EUR_2017
rs921943	T	C	Height	Neale B	UKBB	European	2017	Neale-B_UKBB_EUR_2017
rs921943	T	C	Sitting height	Neale B	UKBB	European	2017	Neale-B_UKBB_EUR_2017

SNP, single-nucleotide polymorphism; EA, effect allele; OA, other allele.

Supplementary Table S3 The characteristics of blood and toenail selenium level associated SNPs.

SNP	Chr	pos	MAF	Gene	EA	OA	R^2	*F*
rs10944	5	78385845	0.49	BHMT2	T	G	0.034	2695.817
rs11951068	5	78304314	0.07	DMGDH	A	G	0.009	738.756
rs1789953	21	44482936	0.14	CBSL	T	C	0.006	478.192
rs3797535	5	78300397	0.08	DMGDH	T	C	0.013	1035.054
rs567754	5	78416416	0.34	BHMT	T	C	0.018	1409.291
rs672413	5	78278229	0.32	ARSB	A	G	0.011	868.591
rs6859667	5	78745042	0.96	HOMER1	T	C	0.010	775.052
rs705415	5	78291960	0.14	DMGDH	T	C	0.010	749.791
rs921943	5	78316476	0.29	DMGDH	T	C	0.035	2765.733

SNP, single-nucleotide polymorphism; Chr, chromosome; pos, position based on GRCh37/hg19; MAF, minor allele frequency; EA, effect allele; OA, other allele.

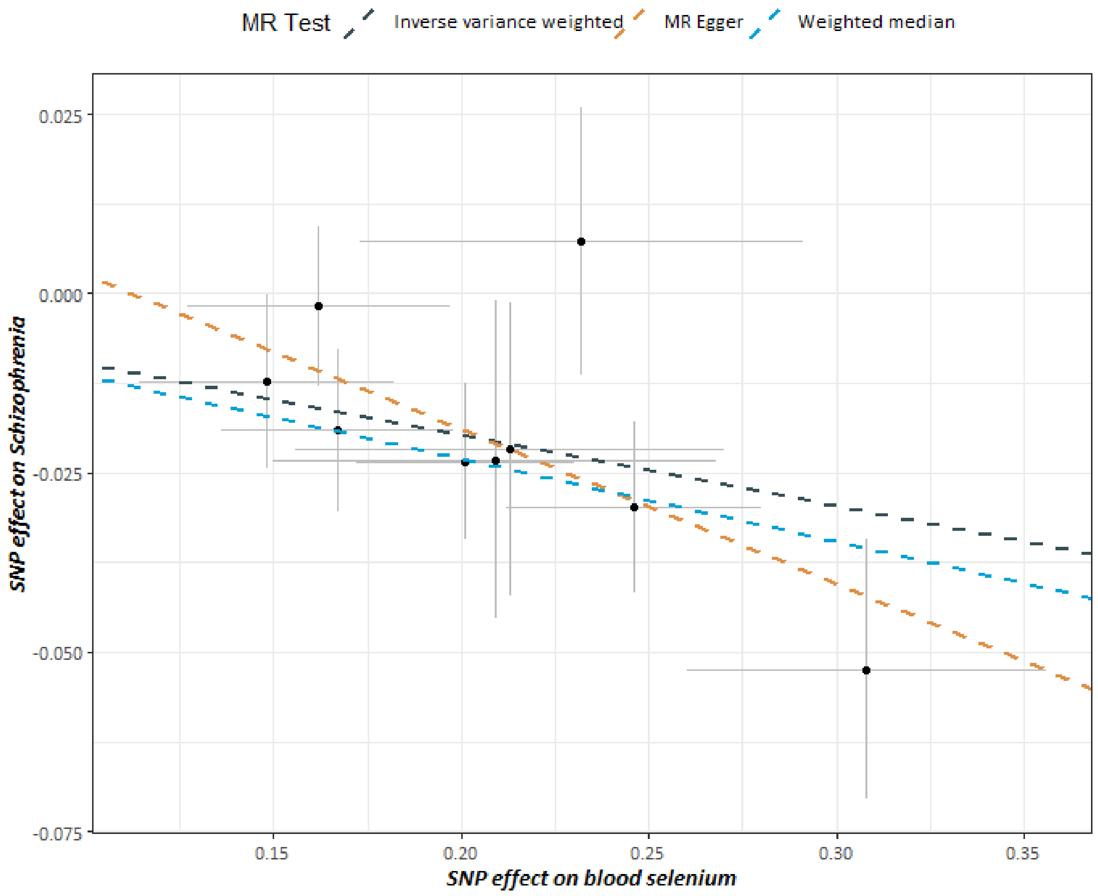

Supplementary Figure S1 Scatter plot of the effect estimates of IVs on blood selenium and risk of schizophrenia.

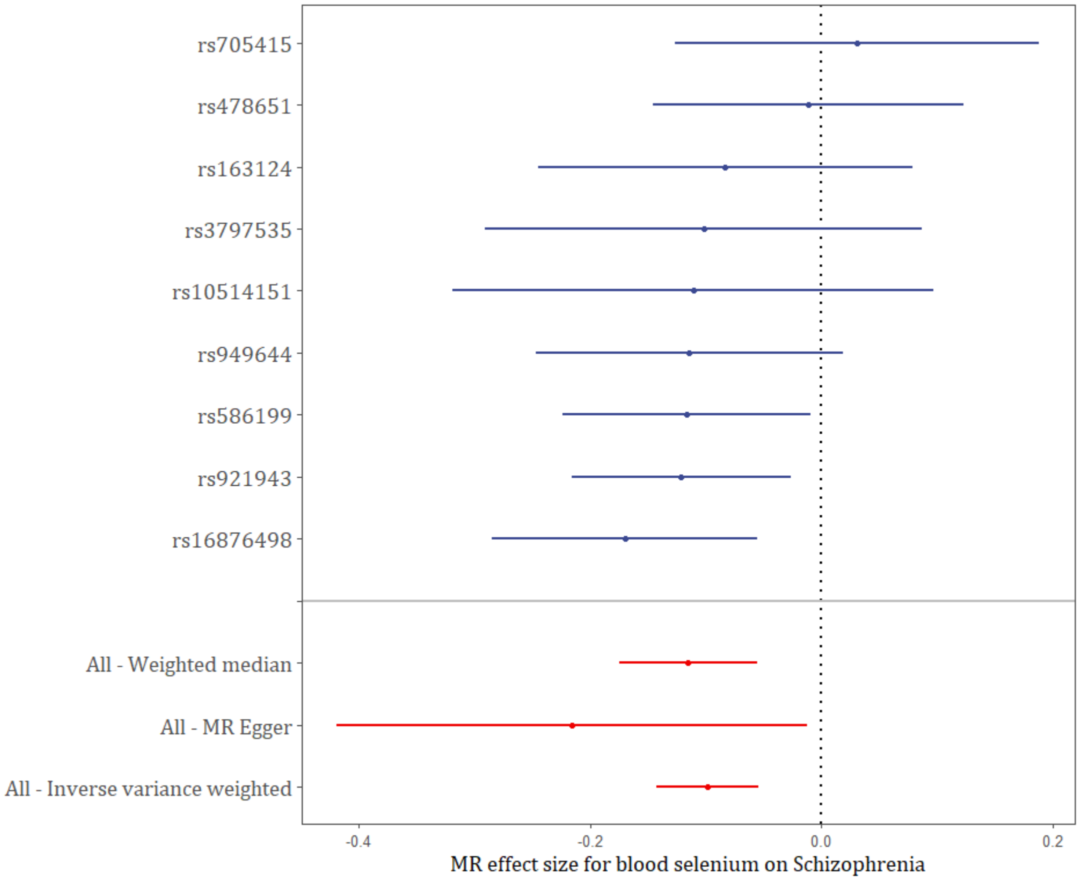

Supplementary Figure S2 Forest plot combing the single SNP effect estimates of blood selenium on the risk of schizophrenia.

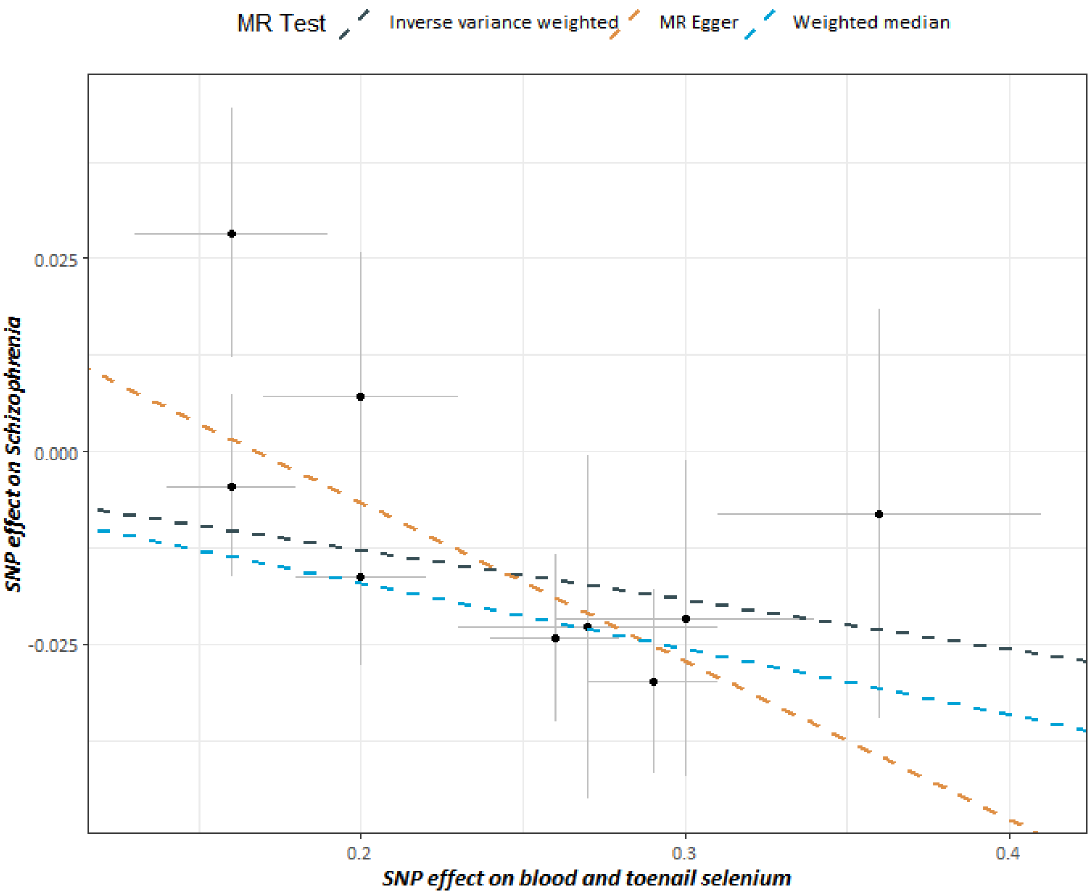

Supplementary Figure S3 Scatter plot of the effect estimates of IVs on blood and toenail selenium and risk of schizophrenia.

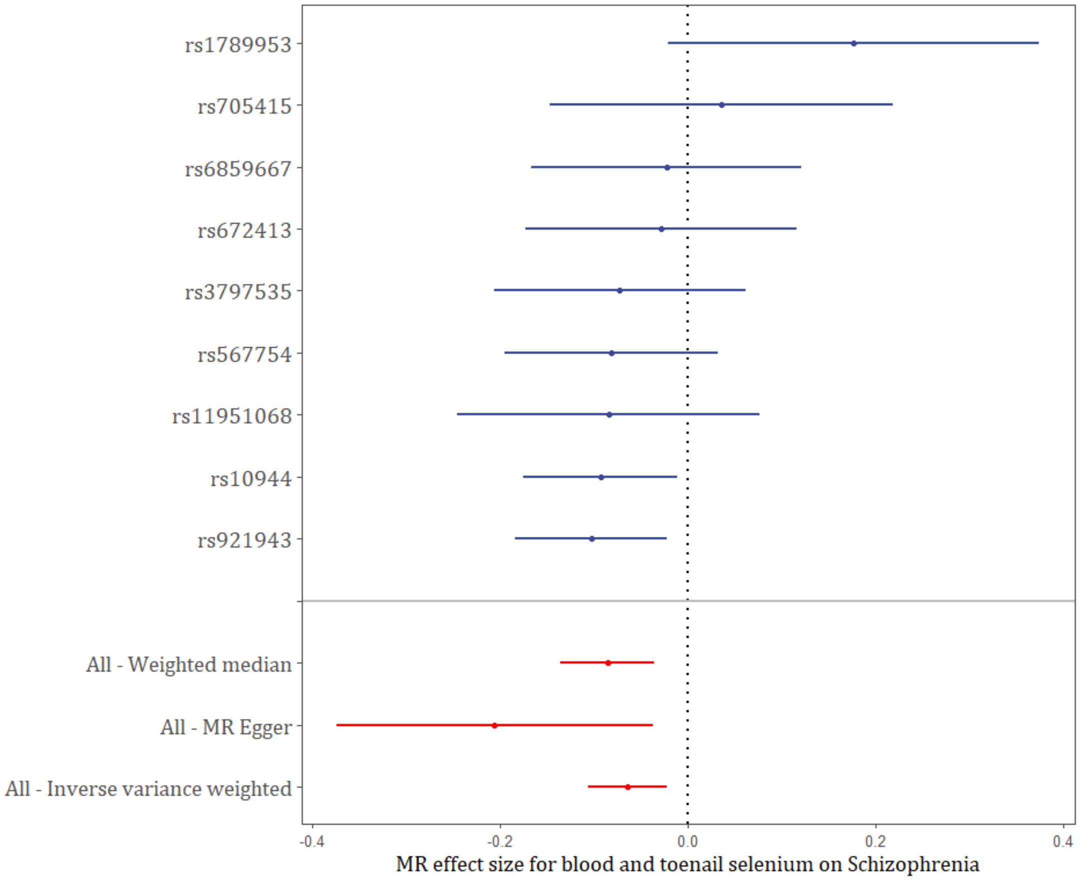

Supplementary Figure S4 Forest plot combing the single SNP effect estimates of blood and toenail selenium level on the risk of schizophrenia

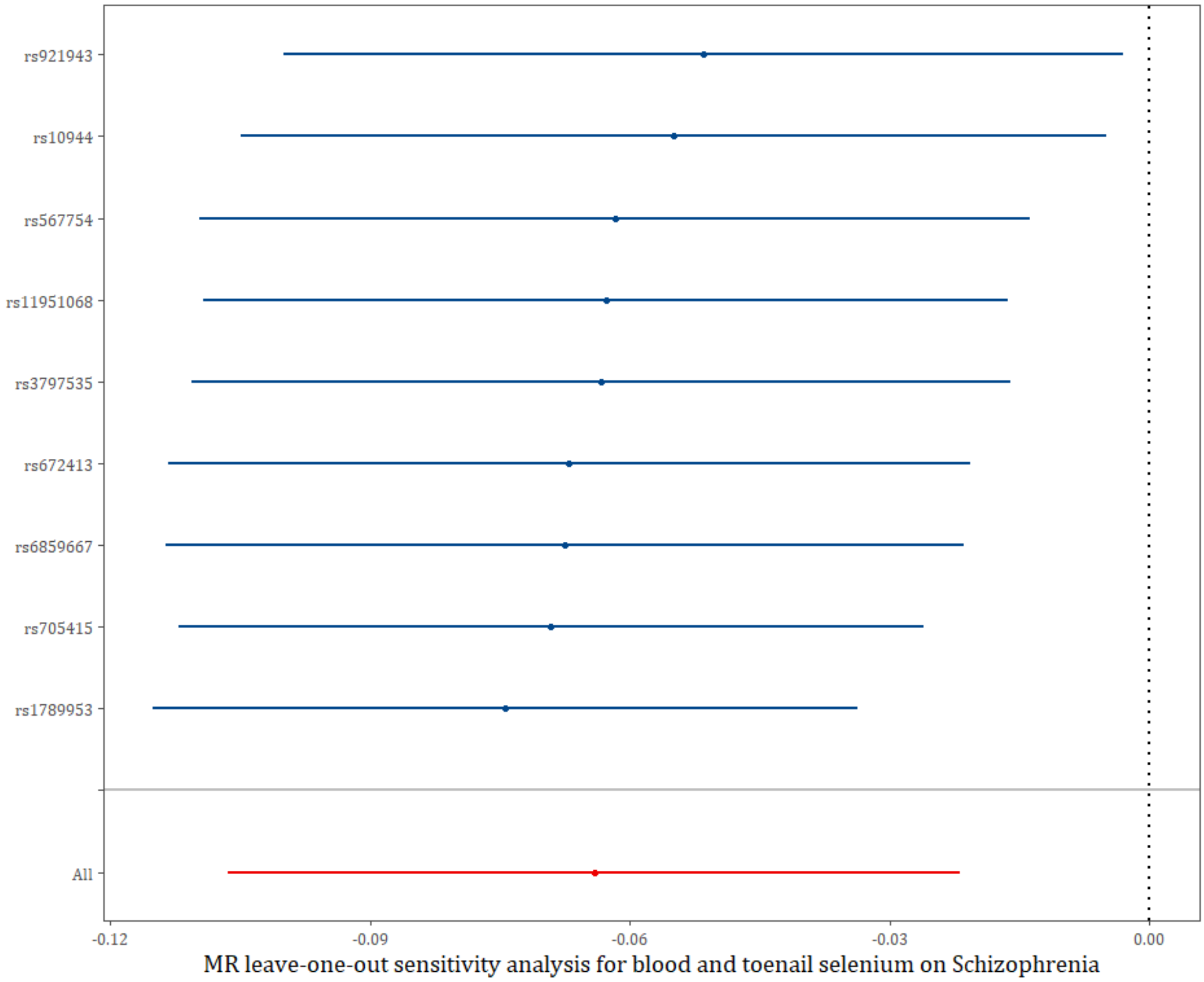

Supplementary Figure S5 The leave-one-out analysis of genetic variants of blood and toenail selenium level on schizophrenia.
